# CAR T Cells in Solid Tumors: Blueprints for Building Effective Therapies

**DOI:** 10.3389/fimmu.2018.01740

**Published:** 2018-07-27

**Authors:** Hannah M. Knochelmann, Aubrey S. Smith, Connor J. Dwyer, Megan M. Wyatt, Shikhar Mehrotra, Chrystal M. Paulos

**Affiliations:** ^1^Department of Microbiology and Immunology, Medical University of South Carolina, Charleston, SC, United States; ^2^Department of Dermatology and Dermatologic Surgery, Medical University of South Carolina, Charleston, SC, United States; ^3^Department of Surgery, Medical University of South Carolina, Charleston, SC, United States

**Keywords:** chimeric antigen receptor, T cell, solid tumor, adoptive cell transfer, checkpoint

## Abstract

Genetic redirection of T lymphocytes with chimeric antigen receptors (CARs) has soared from treating cancers preclinically to FDA approval for hematologic malignancies and commercial-grade production scale in under 30 years. To date, solid tumors are less susceptible to CAR therapies and instead have been treated more successfully with immune checkpoint blockade or tumor-infiltrating lymphocyte therapy. Here, we discuss the current challenges in treating solid tumors with CAR T cells, and the obstacles within the host and tumor microenvironment hindering their efficacy. We present a novel three-pronged approach for enhancing the efficacy of CAR T cells whereby a single infusion product can synergize the power of an optimal CAR construct, a highly potent T cell subset, and rejuvenate the endogenous immune response to conquer therapeutically-resistant solid tumors.

## Introduction

Healing is a matter of time, but it is sometimes also a matter of opportunity—Hippocrates

The interactions between antigen-presenting cells and T cells enable high fidelity host protection against foreign pathogens and malignant cells. T cells have unparalleled ability to not only respond to these antigens but also to formulate memory, permitting a rapid and robust response upon future challenge against the same antigen. In terms of cancer, this potentially means long-term protection against recurrence of tumor cells expressing those antigens. Tumors can express antigens that are rapidly recognized by T cells, where mutations of self-antigens or germline cancer antigens differ sufficiently from normal antigens, or those that are less robustly detected, such as overexpressed self-antigens or differentiation antigens expressed by tissue from which the tumor originates (1). As a result, tumors that are more similar to normal cells, or those with highly immune-suppressive qualities, escape surveillance, permitting their outgrowth and potential to cause great harm. Many technological advances have created opportunities for cancer immunotherapists to bolster the power of T cells against cancer through reeducation and intelligent design to overcome the evasive barriers established by solid tumors. Perhaps immunotherapy represents one such opportunity posited by Hippocrates—a chance for intervention that could heal cancer patients much more effectively than time itself.

Adoptive cell transfer (ACT) comprises one of two arms of immunotherapy and involves *ex vivo* enrichment of tumor-specific cells, expansion to large numbers, and reinfusion into the patient to specifically target and kill cancer cells. ACT is conducted *via* two approaches: (1) naturally arising T cells that infiltrate the tumor—called tumor-infiltrating lymphocytes (TILs)—can be expanded *ex vivo* from the malignant site or (2) non-therapeutic endogenous lymphocytes obtained from the peripheral blood can be rendered tumor specific *via* genetic redirection with a T-cell receptor (TCR) or chimeric antigen receptor (CAR). The second arm of immunotherapy includes immune checkpoint blockade (ICB), where enhancing priming or rejuvenating exhausted T cells can render a functional, albeit often transient, antitumor state. This review will focus on CAR T cell therapies and how future CARs may work synergistically with other immunotherapies to drive long-lasting cures in patients.

The CAR combines a single chain variable fragment (scFv) ectodomain that can target an antigen of choice with an endodomain comprised of the CD3ζ TCR signal and additional costimulatory domain. Its first use by Kuwana et al. and Gross et al. in the late 1980s revealed that redirection of a T cell with this receptor could induce antigen recognition without the major histocompatibility complex (2, 3). CAR-redirected T cell therapies have been successful in hematologic malignancies but are less effective in treating the majority of patients with solid tumors to date. For solid tumors, immunotherapy based in TIL generation or ICB has been more successful. Conceivably, harnessing a CAR therapy with mechanisms of success from TIL and ICB therapies is a logical approach to overcome the obstacles preventing their effective regression of solid tumors. This review will discuss the current status of CAR therapies for solid tumors and outline a three-pronged approach to enhance these therapies against treatment-resistant cancers based on lessons learned with adoptive immunotherapy.

## Destinations of Car T Cell Immunotherapy

The ability to harness an immune response against cancer through ACT or ICB has reinvigorated cancer therapies by improving outcomes in patient populations previously resistant to conventional treatment. Genetic redirection of T cells with specificity against a chosen antigen provides theoretical opportunity to invoke long-term immunity, but with varied results based on type of tumors targeted (4, 5). Herein, we will review recent triumphs of CAR T cells against B cell hematologic malignancies, and the challenges currently preventing similar efficacy in treatment of aggressive solid tumors.

### Success in Hematologic Malignancies

Since 2010, numerous clinical trials have demonstrated the ability of CAR T cells directed against CD19 to promote clinical responses in acute lymphoblastic leukemia (ALL) (6–10), diffuse large B cell lymphoma (DLBCL) (11–13), chronic lymphocytic leukemia (CLL) (14, 15), and other B-cell non-Hodgkin lymphomas (16, 17) with remissions of up to 90% in some of these cases. Because CD19 is expressed ubiquitously in the B cell lineage, targeting CD19 ablates this cell compartment in patients, though sparing of some plasma cells with long-term humoral immunity is possible (18). Fortunately, B cell aplasia can be treated with immunoglobulins to prevent infections, making this a serious but manageable on-target/off-tumor toxicity (19). As a result of excellent responses in patients refractory to standard of care therapies, two constructs of CD19-CAR T cells have been granted FDA approval. Tisagenlecleucel (KYMRIAH, Novartis), with the 4-1BB/CD3ζ costimulatory domain, was approved in August 2017 for B-ALL (20) and in May 2018 for DLBCL, and axicabtagene ciloleucel (YESCARTA, Kite Pharmaceuticals), with the CD28/CD3ζ costimulatory domain, was approved for DLBCL in October 2017. Administration of these CAR T cell therapies requires specialized training under the FDA Risk Evaluation and Mitigation Strategies to manage adverse events such as cytokine release syndrome or neurotoxicity. These approvals render CAR T cells the first FDA approved personalized gene therapy and establish a major milestone in the field of cancer immunotherapy.

Unfortunately, the dramatic responses reported in patients with B cell malignancies have not yet been consistently reproduced with analogous therapies for individuals with solid tumors. However, it is important to appreciate that CAR T cell development for patients with solid tumors is still in early stages. The historical progress, current status, and major obstacles facing success of these therapies in conquering solid tumors are discussed below.

### Clinical Challenges in Solid Tumors

While the results of CAR T cells in B cell malignancies are encouraging, treatment of solid tumors with similar approaches has yielded less favorable results. Similar to therapies for hematologic malignancies, the difficulty in initial design begins with constructing the CAR against an antigen expressed in the tumor—but not in normal tissue—to bolster efficacy while reducing off-tumor toxicity (21). Thus far, clinical trials with CAR T cells in solid tumors have demonstrated severe toxicities since the targeted antigens are often not completely foreign to the host, and even low expression in distant tissues can instigate devastating effects in the presence of a potent T cell therapy (22, 23). Several examples of off-tumor responses in clinical trials are as follows: in renal cell carcinoma, targeting carbonic anhydrase IX (CAIX) led to liver toxicity in 4/8 patients in 2/3 cohorts due to basal expression of CAIX on bile duct epithelium even with low doses of CAR T cells (24, 25). CAR T cells engineered against ERBB2 given in a high dose to a patient with metastatic colorectal cancer caused multi-organ failure with acute pulmonary toxicity due to antigen expression on lung epithelium (26). This resulted in death of the patient within 5 days post-transfer of the cellular product (26). Similarly, a trial for gastrointestinal tumors with CEACAM5-CAR T cells was closed due to poor efficacy and persistence of cells, in addition to toxicity from expression of the targeted antigen on lung epithelium (27). Careful consideration of target antigens is therefore warranted so that a balance between safety and efficacy can be maintained for patients.

Some antigens specific to tumors have been identified that result in more limited off-tumor effects, but many of these targets for CAR T cells have mediated poor clinical efficacy in patients. Several studies using HER2-based CAR in sarcoma (28), mesothelin-specific CAR in mesothelioma and pancreatic cancer (29–31), carcinoembryonic antigen for colorectal cancer (32), EGFRvIII in glioblastoma (33), and α-folate receptor in ovarian carcinoma (34) have shown safer toxicity profiles but yield no better treatment outcomes than stable disease in most cases. Furthermore, similar to CD19^+^ B cell malignancies (9, 35), solid tumors treated with therapeutic CAR T cells can undergo antigen escape due to selection pressure favoring tumor cells lacking the targeted antigen. High frequency of EGFRvIII loss in glioblastoma patients, though indicating the CAR T cells are potent against their target, highlights the importance of heterogeneity in antigen targeting for future solid tumor CAR treatments to be successful (36). Despite these challenges, there has been recent success with CAR T cell therapy in glioblastoma. Localized delivery of CAR T cells engineered against IL-13Rα for recurrent glioblastoma resulted in an objective response lasting 7.5 months in one patient with several intracranial and spinal tumors (37, 38). Obtaining responses in such aggressive, end-stage cancers emphasizes the vast potential for CAR T cell therapies and the importance of their future development.

**Figure 1:**
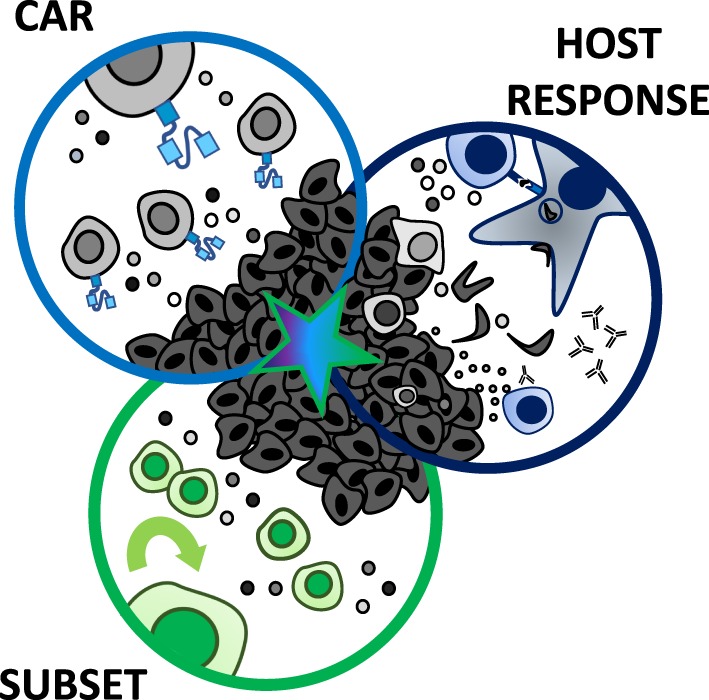
Three-pronged approach to improve chimeric antigen receptor (CAR) T cell therapies in solid tumors. A multi-faceted attack on solid tumors resistant to standard CAR T cell therapies may best augment their efficacy in clinical trials. The ultimate CAR T cell therapy should encompass three axes: (1) a CAR with high fidelity targeting of more than one tumor antigen and trafficking capacity, (2) selection of a T cell subset with potent self-renewal and migratory capacity for long-term persistence and immunity, and (3) ability to harness and rejuvenate the host response to tumor neoantigens. A single arm (CAR, subset, or host response) has not been sufficient for long-term responses against aggressive solid tumors to date.

Theoretically, even if the perfect antigen for a solid tumor could be identified and targeted, CAR T cell therapies for solid tumors face further obstacles including poor trafficking to the tumor site (39), as well as limited persistence and proliferation within the host (27, 34, 40–42). Moreover, CAR T cells can be functionally suppressed within the hostile tumor microenvironment (43). These collective hurdles set solid tumor CAR-based therapies apart from liquid tumors (21, 44). The question puzzling the medical community today is how—or if—we can overcome these significant barriers and cure solid tumors with a CAR T cell therapy approach. Reflecting upon these challenges, we hypothesize that the ultimate CAR therapy for solid tumors may be established *via* a three-pronged approach, as illustrated in Figure 1. The most therapeutic strategy should (1) encompass specificity through the CAR construct, (2) select for a T cell subset with enhanced persistence, trafficking, and long-lived memory responses, and (3) synergize with the endogenous host response to neoantigens. We will review our field’s progress on encompassing these three axes thus far and present our blueprint for what may be necessary to combat solid tumors with next-generation CAR-based approaches.

## Evolution of the Car Design

**Figure 2:**
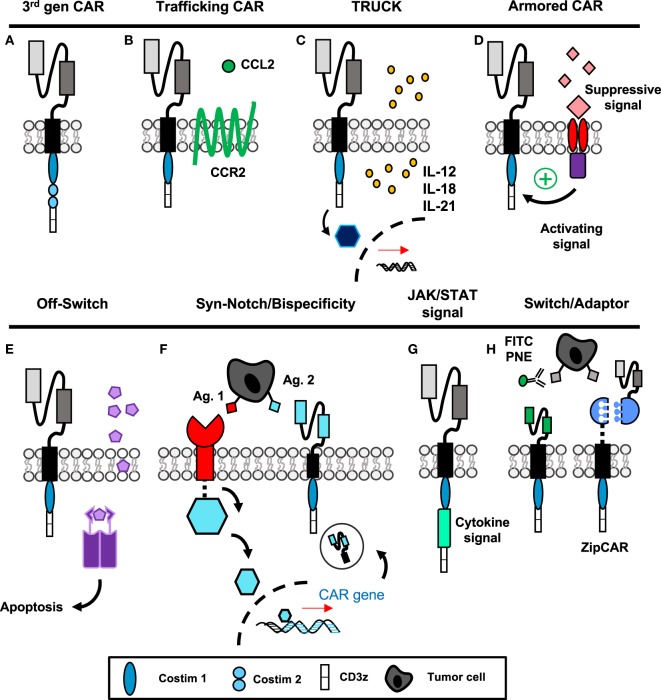
“Fourth-generation” chimeric antigen receptor (CAR) constructs incorporate novel mechanisms to enhance targeted antitumor efficacy. **(A)** The third-generation CAR incorporates the extracellular scFv with intracellular CD3ζ signaling and two tandem costimulatory domains. **(B)** CAR T cells with additional chemokine receptors have improved trafficking to tumors. **(C,G)** T cells secreting additional cytokines or engineered with cytokine signaling domains have enhanced activation and can modulate surrounding microenvironment. **(D)** Armored CARs redirect suppressive signals from the tumor to activating signals to resist exhaustion. **(E)** Suicide genes and **(F)** bispecificity mitigate off-tumor toxicity through the ability to deplete transferred cells or enhance specific targeting to tumors, respectively. **(H)** Switchable CAR targeting *via* adaptor molecules provides versatile opportunity to control CAR activation, specificity, and longevity after transfer of cells. Abbreviations: Ag, antigen; PNE, peptide neo-epitopes.

The first three generations of CAR construct design have evolved to incorporate two activating signals (TCR-signaling domains and costimulation) to enhance functionality of therapies *in vivo* and have been reviewed previously (45–47). Briefly, the first-generation CAR, pioneered by several groups in the late 1980s (2, 3, 48–50), consisted of only the scFv region and CD3ζ intracellular domain. These cells demonstrated poor efficacy and expansion in response to antigen, especially if given without exogenous IL-2 (51). The second-generation CAR includes an additional costimulatory domain while the third generation (Figure 2A) uses two costimulatory domains in tandem (52); both have greatly enhanced efficacy over the first generation. CD28 was incorporated first (53), followed by ICOS (54), OX40 (CD134) (54, 55), and 4-1BB (CD137) (54, 56, 57). While the optimal costimulatory signals are under debate and may depend on the T cell subset itself, 4-1BB signaling has been shown to improve persistence (15, 58) and enhance metabolic fitness and memory potential of CAR T cells over CD28 (59), and the combination of 4-1BB and ICOS appears promising preclinically (60).

Due to a lack of clinically successful CAR therapies in patients with solid tumors, numerous groups have been inspired to design “fourth-generation” CAR constructs incorporating novel mechanisms to improve antitumor activity. These approaches include enhancing migration and efficacy of the engineered cell, as well as the ability to resist immunosuppression and off-tumor toxicity, illustrated in Figure 2 and discussed directly below.

### Enhancing Migration

Tumors that express fewer chemokines often evade host surveillance *via* impairing effector T cell recruitment and infiltration into the tumor (61). Several different chemotherapeutics have been shown to induce CXCR3-ligand and CCL5, which enhance CD8^+^ T cell recruitment and reduce tumor growth (62). One chemokine in particular, MCP-1/CCL2, has been correlated with enhanced CCR2-expressing T cell trafficking when secreted by tumors such as gliomas, neuroblastoma, renal cell carcinoma, and mesothelioma (63). For CAR T cells, *ex vivo* activation protocols can alter expression of chemokine receptors, where those such as CCR2 are frequently downregulated (64). Two groups have shown that forced expression of CCR2 on CAR T cells (Figure 2B) targeting either GD2 in neuroblastoma (65) or mesothelin for malignant pleural mesothelioma (64) enhances T cell infiltration and augments antitumor activity of the transferred cells. In melanoma, poor T cell infiltration within tumor has been correlated with high tumor IL-8/CXCL8 expression (61); therefore, future CAR T cells engineered to express CXCR1 or CXCR2 may also be more efficient at targeting melanoma. As various solid tumors express unique combinations of chemokines, further understanding of these chemokine profiles could aid in the design of novel CAR T cells that can traffic more robustly to the particular cancer they are intended to destroy.

### Augmenting Efficacy

As solid tumors have proven to be formidable foes, CAR T cells fortified with enhanced properties of cytokine secretion or cytokine signaling domains have several unique advantages to overcome limitations of the tumor microenvironment, as depicted in Figures 2C,G. If the T cell produces a cytokine related to cytotoxic effector programming upon ligation of the CAR, autocrine signaling can activate and support the antitumor activity, persistence, and survival of the transferred cells. In addition, tumor-targeting CAR T cells can deliver cytokines to modulate the cancer microenvironment in an advantageous manner to either activate host effectors or hinder host suppressors to bolster memory T cells in the patient long term.

These cytokine-producing “TRUCKs” (T cells Redirected for Universal Cytokine Killing) have shown efficacy when delivering IL-12, IL-15, IL-18, or IL-21 to the tumor microenvironment (Figure 2C) (66). Of particular clinical importance, IL-12-producing CARs were reported to be therapeutic against lymphoma even without preparative lymphodepletion (67), and significantly enhanced efficacy of MUC-16^ecto^ CAR against a preclinical model of ovarian carcinoma (68). IL-12-producing CD8^+^ T cells modulated suppressive host myeloid cells within the tumor microenvironment, and as a result improved therapeutic efficacy (69). In a clinical trial for metastatic melanoma patients, autologous TIL engineered to secrete IL-12 yielded objective responses in lower doses compared with unmodified TILs and without systemic administration of IL-2; however, many of these responding patients developed severe liver toxicities and hemodynamic instability (70). Moving forward, it will be critical to deliver localized and inducible IL-12 production within the tumor microenvironment *via* TILs or CAR T cells to more specifically direct its potency while minimizing risk of unacceptable toxicity. IL-15 production similarly improved survival and proliferation of CAR T cells specific for CD19 in leukemia/lymphoma (71) and IL-13Rα2 in glioblastoma (72), as did membrane-bound IL-15 for CD19^+^ leukemia without significant toxicity (73). Recently, IL-18-producing CAR T cells have been developed. Administration of IL-18 has been shown to augment immunity in solid tumors *via* activating natural killer (NK) cells (74) and is known to induce IFN-γ production from Th1 cells in the presence of IL-12 (75, 76). In CD19^+^ tumors, IL-18-producing TRUCKS improved engraftment and long-term survival of hosts bearing established tumors (77). Importantly, in mouse models of pancreatic carcinoma and metastatic lung adenocarcinoma—classically highly resistant to treatment—Chmielewski and Abken established that IL-18 secretion and autocrine signaling can induce a T-Bet^High^ FoxO1^low^ signature in the CAR T cells and augment tumor infiltration of NKG2D^+^ NK cells, while reducing the frequency of regulatory T cells (Tregs) and suppressive macrophages in the tumor microenvironment (78). While improved proliferation and cytokine production within the host are important to antitumor efficacy, the possibility of cytokine-induced dysregulation of CAR expansion or toxicity highlights the need for a form of safety switch or suicide gene within the CAR (71).

IL-21 is a homeostatic cytokine that has shown promise in preclinical TIL and CAR studies, and may be a desirable future candidate to bolster responses in adoptive transfer clinical trials. Programming CD8^+^ tumor specific lymphocytes *ex vivo* with IL-21 was reported to reduce the activation/exhaustion phenotype of terminally differentiated cells observed after long-term expansion with IL-2 (79). While *ex vivo* cytolytic function of CD8^+^ T cells upon antigenic stimulation was reduced with IL-21 priming, the *in vivo* melanoma regression was greatly enhanced long term compared with CD8^+^ T cells primed with IL-2 or IL-15. Systemic administration of IL-21 also enhanced efficacy of tumor-specific CD8^+^ T cells against melanoma in a preclinical model (80). IL-21 fosters generation of antitumor T cells expressing Tcf7, L-selectin, and Lef1 in the Wnt/β-catenin pathway, inducing a signature of stem-like properties that may support long-lived memory of transferred CAR T cells clinically (79). IL-21 programming of human CAR T cells *ex vivo* was also shown to improve efficacy against CD19^+^ tumors *in vivo* (81). Furthermore, in a direct comparison, CAR T cells producing IL-21 were superior to IL-15- or IL-2-producing CARs against CD19^+^ malignancies (82). Therefore, maintenance of memory characteristics *in vivo* through inducible IL-21 expression in CAR T cells, theoretically also supporting memory of endogenous tumor-specific T cells, may greatly improve the longevity of future CAR therapies for long-lasting curative responses.

Another related application of this concept has been described where the CAR construct encodes a costimulatory domain as well as a cytokine signaling domain for IL-2Rβ (Figure 2G) (83). Therefore, the CAR T cell does not produce the cytokine, but the pathway downstream of the desired signal is activated upon engagement of the scFv fragment with antigen. Unfortunately, this approach is restricted to augmenting the CAR T cell’s efficacy and not the endogenous host response. However, with cytokines like IL-2, which signal to both effector and Tregs, this approach can restrict signal activation to the effector arm of the antitumor response. Collectively, manipulating cytokine production or cytokine signaling has opened new possibilities for generating CARs with desirable traits to bolster their efficacy against tumors and improve immunity of other infiltrating immune cells.

### Evading Immunosuppression: Turning Lemons into Lemonade

Even when CAR T cells successfully invade the tumor, they face a microenvironment rich in suppressor cytokines, such as TGF-β and IL-4, and inhibitory molecules including PD-L1 that poise the cancer to escape immunity. To make these limitations advantageous, tumor immunologists are now redirecting TCR or CAR-specific T cells with additional domains that either (1) limit suppressive signaling or (2) convert suppressive signals into activating signals, thus “armoring” T cells against the suppressive elements of the tumor (Figure 2D). The earliest studies using this approach were with Epstein–Barr virus-specific T cells engineered with a dominant negative mutation of the TGF-β receptor, which allowed tumor-specific T cells to resist suppression by the tumor-derived TGF-β (84). Likewise, PSMA-specific CAR T cells for prostate cancer engineered with a dominant negative TGF-β receptor demonstrated enhanced proliferation post-transfer and are now being used in clinical trials (NCT03089203, Table 1) (85). New studies with CAR T cells have used a chimeric cytokine receptor that binds IL-4, a cytokine that suppresses immunity, *via* an ectodomain but transmits a therapeutic IL-7 signal *via* the endodomain. When IL-4 binds the receptor, instead of the anti-inflammatory STAT6 translocation, the IL-7 pathway phosphorylates STAT5 and polarizes the cell toward an inflammatory Th1 response (86). Similarly, a PD-1/CD28 chimeric switch receptor has been designed to convert an exhaustive stimulus into a costimulatory signal; this construct was shown to enhance cytokine production and *in vivo* efficacy in the presence of PD-L1^+^ prostate cancer cells compared with CAR-only transduced cells (87). Two clinical trials are ongoing in China with the use of chimeric switch receptors and are described in Table 1. These advances in T cell engineering may now enable reversal of mechanisms driving CAR T cell failure in solid tumors.

**Table 1:** Clinical trials of fourth-generation chimeric antigen receptor (CAR) T cells in solid tumors.

4th Generation CAR T cells in solid tumors		

ClinicalTrials.gov identification	Trial description	Location(s)
**“Armored” CAR**		
NCT03089203	CAR T cells targeting PSMA for castration-resistant prostate cancer with dominant negative TGF-β receptor	University of Pennsylvania
NCT02937844	Pilot study of autologous chimeric switch receptor modified T cells in recurrent glioblastoma multiforme	Sanbo Brain Hospital Capital Medical University, Beijing, China
NCT02930967	Chimeric switch receptor with PD-L1^+^ recurrent or metastatic malignant tumors	China Meitan General Hospital

**Suicide genes**		
NCT00730613	CAR T against IL-13Ra2 in glioblastoma with Hy/TK suicide switch	City of Hope Medical Center
NCT02992210	4SCAR-GD2 targeting CAR with iCaspase9 domain in refractory solid tumors	Shenzhen Geno-Immune Medical Institute
NCT02414269	Malignant pleural disease treated with Meso-CAR T cells, modified with iCasp9/M28ζ	Memorial Sloan Kettering Cancer Center
NCT01822652	GD-2-CAR T (28-Ox40ζ) and iCaspase9 Suicide safety switch for Neuroblastoma	Baylor College of Medicine
NCT03185468	4SCAR-GS2 with iCaspase9 domain in advanced/metastatic urothelial carcinoma	Shenzhen Geno-Immune Medical Institute

**Antibody-producing CAR T cells**		
NCT03179007	CTLA-4/PD-1 antibody expressing MUC-1 CAR T for MUC1^+^ advanced solid tumors	Shanghai Cell Therapy Research Institute
NCT03182803	CTLA-4/PD-1 antibody expressing mesothelin-CAR T for Meso^+^ advanced solid tumors	Shanghai Cell Therapy Research Institute
NCT03182816	CTLA-4/PD-1 antibody expressing EGFR-CAR T for EGFR^+^ advanced solid tumors	Shanghai Cell Therapy Research Institute
NCT02862028	PD-1 antibody expressing CAR T cells for EGFR family member positive advanced solid tumor (liver, lung, stomach)	Shanghai International Medical Center, Shanghai, China
NCT02873390	PD-1 antibody expressing CAR T cells for EGFR family member positive advanced solid tumor	Ningbo Cancer Hospital, Zhejiang, China
NCT03030001	PD-1 antibody expressing mesothelin-specific CAR T cells for meso^+^ malignant tumors (recurrent or refractory)	Ningbo Cancer Hospital, Zhejiang, China
NCT03170141	4SCAR-IgT against EGFRvIII on glioblastoma multiforme, producing PD-1 and PD-L1 antibodies	Shenzhen Geno-Immune Medical Institute

### Mitigating Off-Tumor Toxicity

Finally, CAR T cell depletion in patients experiencing uncontrolled toxicity and engineering approaches to enhance specificity to solid tumor antigens are two methods to reduce severe toxicities previously discussed. Suicide genes to deplete CAR T cells, incorporation of epitopes for antibody neutralization, and logic gate control of CAR T cell function have been described. The first examples of suicide genes involved use of HSV-thymidine kinase, which converts ganciclovir into a toxic metabolite (88). However, the problem with this approach is that the response is slow (89) (several days) and the viral proteins themselves may be immunogenic leading to rejection of the cells (90). In recent development, the inducible-caspase 9 system armors the CAR with a homodimer iCasp9 domain that dimerizes upon administration of a small molecule (Figure 2E) (89). Dimerization leads to cleavage of caspase 3 and apoptosis of the CAR T cells. Several clinical trials are now incorporating such safety switches into their CAR programs, which are outlined in Table 1. In addition, incorporating epitopes like RQR8/CD20 into the CAR construct provides a target for their depletion with antibodies such as rituximab (91). This approach depletes the majority of CAR T cells within a few hours (91). As rituximab is widely used clinically, this is a non-toxic and relatively inexpensive method for rapid deletion of CAR T cells in case of severe toxicity. Though protective against severe toxicities, the iCasp9 and antibody-directed depletion approaches do not differentiate cells causing off-tumor side effects from cells with therapeutic efficacy, which could result in loss of any clinical benefit against tumors.

To improve the discriminatory nature of strategies used to reduce toxicity, design of CAR T cells equipped with tetracycline-inducible systems or AND/NOT Boolean logic gates permit enhanced control over effector responses and improved sensing of tumor targets. Sakemura and colleagues established a Tet-on inducible system for CD19^+^ malignancies, where administration of a tetracycline turns on CAR expression—useful for a period of heavy tumor burden—while withdrawal of the drug ceases CAR expression but permits survival of the cell—important for periods of off-tumor toxicity (92, 93). Boolean logic gates aim to prevent toxicity while maintaining efficacy, rather than irreversibly deleting CAR T cells that are toxic against both tumor and host. First, AND gates require a combination of antigens for full T cell activation. In prostate cancer, Kloss and colleagues demonstrated that high affinity CAR and chimeric costimulatory receptors targeting two antigens, such as PSMA and PSCA, leads to eradication of cells bearing either target (94). However, with low affinity receptors, activation of one receptor was not sufficient for full T cell activation, making the presence of both antigens necessary for activation (94). Wendell Lim and colleagues have pioneered the use of syn-Notch receptors in CAR T cells where engagement of a tissue-specific antigen by a surface receptor induces transcription of a CAR against a tumor-specific antigen (Figure 2F) (95–97). These approaches allow increased sensitization to tumor cells and reduced toxicity against healthy tissues bearing only one of the targeted antigens. Alternatively, NOT gates employ receptors that prevent T cell activation. For example, the iCAR developed by Fedorov et al. has two receptors with opposite functions: first, a receptor for an off-target antigen such as one found on healthy tissue signals the inhibitory cascade downstream of CTLA-4 or PD-1, while a second tumor-specific receptor signals CD3ζ and costimulation for T cell activation (98). Therefore, CAR T cells can be designed to discriminate between on- and off-tumor targets without compromising survival of the transferred T cells. With these novel CAR T cell designs, toxicities can be managed without loss of antitumor function, though indication of each approach may vary depending upon the type of tumor and immunogenicity of the antigens targeted.

While combinatorial or logical sensing may enhance specificity of CAR T cells to tumor targets in the future, the search for antigens specific for tumors remains an important ongoing approach. Self-antigens are frequently modified through processes such as glycosylation as they undergo mutagenesis and cells experience malignant transformation (99). CAR T cells targeting glycosylated self-antigens in the tumor are potent against several solid tumor types and minimally toxic to the host due to the specificity of glycosylation sites for the tumor (100). Overall, a better understanding of how self-antigens are modified in tumors may represent a simpler approach to achieve high potency and low toxicity clinically.

### Remote-Controlled CARs

Very recently, CAR T cells active only in the presence of a soluble, inert adaptor molecule have been brought to life in preclinical systems (Figure 2H). Early studies incorporated CAR T cells engineered for specificity against FITC (101) or PNEs (102), which are linked to antibodies specific for antigens on tumor. Recently, “SUPRA” (split, universal, and programmable) CAR T cells were developed where a “zipCAR” domain links an intracellular costimulatory domain and an extracellular leucine zipper (103). This zipper can be targeted with a complementary zipper fused to an scFv region to render the SUPRA CAR T cell tumor specific. These approaches would be particularly useful for generating universal CAR T cells for various tumors; adaptor molecules could be designed for tumor specificity and would provide options for altering specificity post-adoptive transfer, key for situations of selection pressure and antigen escape. The feasibility and speed of developing a new adaptor with specificity for tumors is likely to be much greater than generating a new, personalized CAR T cell product.

As collectively revealed in Figure 2, the scientific community’s response to challenges in treating solid tumors has been robust and impressive. Indeed, many opportunities now exist for design of future clinical trials incorporating more specialized CAR constructs. However, since persistence of T cells and a long-lasting memory response are ideal for a successful therapy, it is likely that the quality of the lymphocyte itself is as important for building a better CAR to target the antigen. Consequently, we will next discuss what is known about the optimal properties of a T cell for adoptive transfer and future implications of their clinical use in patients with solid tumors.

## Beyond the Car: Pursuit of the Optimal T Cell

*Ex vivo* manipulation of T cells provides a unique opportunity to select the most highly therapeutic cells before transfer, including generation of CD8^+^ lymphocytes with a distinct memory lineage or polarized CD4^+^ helper T cell subsets. Despite the advantages of precisely defining the most effective infusion product composition through cell sorting, most clinical trials of CAR T cells to date infuse bulk products in efforts to transfer large numbers of cells (104). One recent clinical trial at Fred Hutchinson used this selective approach by infusing a 1:1 ratio of CD4^+^ and CD8^+^ cells with a central memory (CM) signature to treat patients with B-ALL; however, only 16 of the 30 patients had enough CD8^+^ T_CM_ cells in the peripheral blood to meet their minimum criteria to enrich this population (105). As technologies advance to permit more efficient T cell purification, so will the feasibility of selecting the optimal T cells for achieving long-term cures in patients. By enriching cell subsets with greater potency, reliance on large doses of T cells may become obsolete. Highlighted below are current novel ways in which investigators are generating T cell subsets with enhanced properties for ACT.

### CD8^+^ Memory Subsets

Debate exists about which memory CD8^+^ T cell subset is ideal for sustaining durable responses to cancer. Some investigators believe that effector CD8^+^ T cells that secrete heightened IFN-γ are more effective against tumors while others argue that less-differentiated or even naïve CD8^+^ T cells are the most ideal lymphocytes to foster long-lasting immunity (106, 107). Therefore, we review previous work defining the role of CD8^+^ T cell differentiation and memory in the context of adoptive T cell transfer therapy.

**Figure 3:**
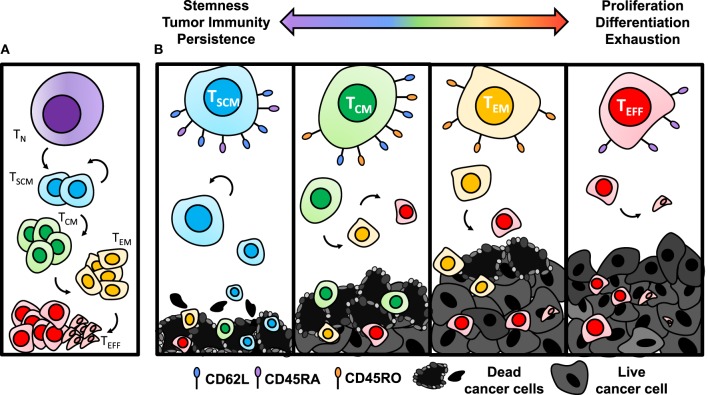
Antitumor efficacy of memory CD8^+^ T cell subsets diminishes with differentiation. **(A)** Once activated with cognate antigen, CD8^+^ T cells progressively differentiate from stem-cell memory (SCM), with the highest capacity of self-renewing properties, to central memory (CM), effector memory (EM), and finally to terminal effector (EFF) phenotypes. **(B)** Antitumor immunity of T_SCM_ cells is enhanced due to establishing long-term memory responses to tumor antigens and heightened ability to persist. As cells become more differentiated through the T_CM_, T_EM_, and T_EFF_ stages, they lose capacity for self-renewal and become exhausted, resulting in poor antitumor immunity.

When activated with a cognate tumor antigen, CD8^+^ T cells differentiate into a short-lived effector phase poised with cytotoxicity against their target. The exact mechanism of this differentiation pathway remains under dispute and two differentiation models have been proposed. One model suggests that naïve cells differentiate directly into the effector phase, followed by de-differentiation into long-lived memory cells. New evidence supporting this model is highlighted by the ability of an individual cell to lose and regain expression of L-selectin without cell division (108). A second model, known as the linear differentiation model, suggests that naïve cells are programmed into T_SCM_ [stem-cell memory (SCM)], T_CM_ [central memory (CM)], and T_EM_ [effector memory (EM)] subsets with varied capability of responding to antigenic rechallenge, terminating with differentiation into effector cells (Figure 3A) (109, 110). Recent epigenetic findings add to this body of literature supporting the linear model of T cell differentiation by showing that after priming, the histone methyltransferase Suv39h1 silences memory genes to direct CD8^+^ T cells into the effector phenotype (111). Without Suv39h1, the memory subsets are preserved after activation while generation of effector subsets is impaired, suggesting that memory phenotypes are enriched before effector phenotypes (111). For a patient in complete remission from CLL after CD19-CAR T therapy, CAR integration into the tumor suppressor gene, *TET2*, resulted in robust clonal proliferation of CAR T cells with a predominantly CM phenotype (112). While this particular integration site was not by design, this clinical example highlights the intricacy of memory differentiation and the implications of driving the T cell toward a particular memory phenotype on patient outcomes. This suggests that epigenetic or genetic manipulation of T cells *ex vivo* could be a novel approach to control memory differentiation of cells and generate a more therapeutic product before transferring cells into patients.

The antitumor efficacy of adoptively transferred memory subsets has been shown to progressively worsen as cells expand logarithmically and often approach the T_EFF_ phase (Figure 3B) (106, 113, 114). By contrast, T_SCM_ cells, characterized by CD45RO^−^CD45RA^+^CD95^+^CD62L^+^ expression (107), were most potent in a direct comparison of human meso-CAR-engineered memory subsets due to their enhanced proliferative capacity and survival (110). In addition, T_SCM_ cells have the ability to self-renew across several cell divisions when reactivated (110). Following the path of differentiation, tumor specific T_CM_ cells are traditionally more effective for long-term regression of established solid tumors than T_EM_ (114), while all memory subsets are superior to T_EFFs_ (113).

**Figure 4:**
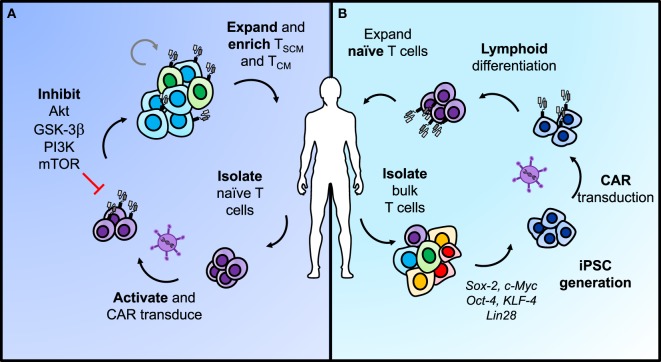
Two approaches for generating less-differentiated T cells after *ex vivo* expansion for adoptive cell transfer. **(A)** Naïve T cells sorted from peripheral blood can be activated and transduced with chimeric antigen receptors (CARs) for antigen specificity. Adding pharmacologic inhibitors of AKT, GSK-3β, PI3K, or mTOR to the T cell culture helps retain cells in a less-differentiated state as they expand. This approach can enrich T_SCM_ and T_CM_ phenotypes in CAR T cells from naïve populations before adoptive transfer to enhance long-term immunity. **(B)** Differentiated T cells can be reprogrammed with stem-like qualities using iPSC technology. In brief, bulk T cells are isolated from the blood, programmed into iPSCs, and transduced with a CAR before lymphoid differentiation into naïve T cells. The most efficient approaches for lymphoid differentiation into naïve phenotypes are still under development.

As a result of finding that less-differentiated memory cells are superior in regressing tumors in ACT models, there is now a growing clinical interest in the ability to expand T cells to large numbers for ACT, while concomitantly inhibiting phenotypic differentiation to foster more stem-like features and enhanced potency against tumors. One approach to accomplish this objective includes targeting downstream of the IL-2 pathway during *ex vivo* expansion through inhibiting subunits of GSK-3β (115), AKT (116), and PI3K (Figure 4) (117). GSK-3β inhibition was shown to bolster Wnt/β-catenin signaling and maintain the T_CM_ phenotype with *ex vivo* expansion, thus improving efficacy of infused antitumor CD8^+^ T cells (115). Inhibition of AKT (AKT inhibitor VIII) (116) or the p110δ subunit of PI3K (Idelalisib/CAL-101) (117) *ex vivo* were also two strong approaches to enrich the frequency of T_CM_ cells in infusion products and improve ACT with CAR-engineered cells for leukemia and mesothelioma models, respectively. However, when directly compared in a transgenic model of melanoma, CAL-101 improved persistence of CD8^+^KLRG1^lo^CD62L^hi^ cells in the peripheral blood and significantly enhanced tumor regression compared with AKTi (117). CAL-101 has also been shown to improve antitumor efficacy of Th17 cells by enhancing the proportion of T_CM_ cells and reducing Tregs in culture (118). These reports reveal that adding small molecules to cultures can propagate T cells with a stem-like memory signature. This approach presents a simple and translatable way to improve both the quality and longevity of antitumor responses.

An alternate approach to generating more naïve-like T cells for ACT involves genetic reprogramming of induced pluripotent stem cells (Figure 4) (iPSCs). Theoretically, reprogramming T cells in this manner poses the opportunity to de-differentiate terminally exhausted tumor- or neoantigen-specific T cells, such as found in a TIL culture, into “younger” more memory-like cells, while retaining their rearranged TCR (119). Early reports on this concept showed the feasibility of generating iPSCs from peripheral blood mononuclear cells *via* induction of Oct4, Sox2, Klf4, c-Myc, and Lin28 (120–122). To move this approach into CAR T cell therapies, Sadelain and colleagues engineered peripheral T lymphocyte-derived iPSC cells to express a CD19-CAR, and subsequently differentiated them back into the lymphoid lineage (123). However, upon phenotypic analysis, they were genetically more closely related to innate γδ T cells and functionally demonstrated weaker antitumor efficacy compared with the desired αβ T cells normally used in ACT (123). This process can also take up to 2 months to generate these CAR T cells, making the time investment on par with or even greater than expanding TIL *ex vivo* (123). Other attempts to program iPSCs down the lymphoid pathway *in vitro* have resulted in abnormal T cell development due to the absence of thymic selection or have generated T cells with effector-like phenotypes (123–125). To generate a potent response against tumors, the αβ^+^ TCR indicative of a more natural T cell is required.

In response to this need, the Restifo lab devised a new approach for generating tumor-specific T cells from iPSCs *in vitro* with a phenotype closer to endogenous, thymic-derived T cells (126). Their 3D thymic culture system generated tumor specific CD8αβ^+^ naïve-like T cells that regressed melanoma and prolonged survival comparably with *bona-fide* naïve T cells obtained from the pmel-1 transgenic mouse spleen (126). This new approach is exciting as it may permit generation of a more robust supply of CAR-engineered naïve-like T cells to mediate long-term cures in patients whose peripheral T cells were previously dysfunctional. Moving forward, inhibition of memory differentiation pathways in *ex vivo* culture and further developments in the feasibility of genetically reprogramming iPSCs will support generation of memory-like CD8^+^ subsets with enhanced antitumor properties, thereby improving patient outcomes.

### CD4^+^ T Cell Subsets

**Figure 5:**
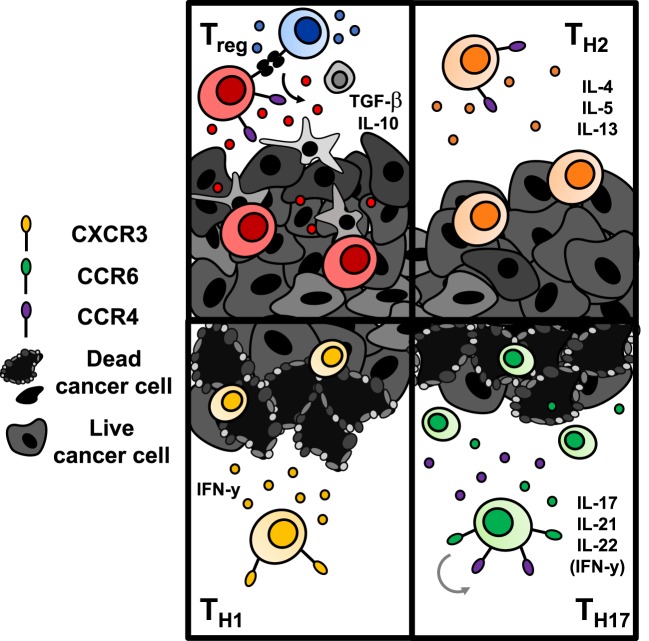
Antitumor immunity of CD4^+^ T cells is dependent upon the subset to which they are polarized. Regulatory T cells (Tregs) (top left) and Th2 cells (top right) are classically tumor promoting. Tregs downregulate effector T cell responses *via* secretion of suppressive cytokines or engagement of inhibitory checkpoint molecules like CTLA-4 or TIGIT. Th2 cells secrete suppressive cytokines that hinder a Th1-mediated antitumor response. Conversely, transfer of Th1 cells (bottom left) and Th17 cells (bottom right) enhance antitumor responses. Th1 cells produce IFN-γ and enhance CD8^+^ cell-mediated immunity. Th17 cells produce proinflammatory cytokines that have controversially been implicated in carcinogenesis; however, adoptive transfer of Th17 cells has shown robust immunity in several solid tumors. Transferred Th17 cells have stem-like self-renewal capabilities and enhanced persistence long term over Th1 cells.

While ACT with CD8^+^ T cells has been more thoroughly studied, the impact of CD4^+^ T helper cells on tumor immunity has recently emerged both preclinically and clinically (127, 128). This body of work indicates that CD4^+^ T lymphocytes may play a key role in enhancing cancer immunotherapy. Since CD4^+^ T cells classically support CD8^+^ T cell activation and proliferation through cytokine secretion, an infusion product containing only CD8^+^ cytotoxic T cells, as is used frequently in the clinic, may show poor persistence simply due to flawed design. Recently, adoptive transfer of a CD4^+^ dominant T cell product resulted in tumor regression in a patient with metastatic cholangiocarcinoma (127) and a complete durable remission in a patient with metastatic breast cancer (128). These cases hint that CD4^+^ lymphocytes may be a powerful subset that should not be selected against. Could it be possible that contrary to accepted dogmas, CD4^+^ T cells may be able to lyse tumor cells themselves without reliance on CD8^+^ T cells? The quality of tumor immunity may ultimately depend upon the CD4^+^ subset transferred, and whether these subsets require CD8^+^ T cells to exert antitumor effects is unclear and will be discussed further below. Herein, we will examine the role of CD4^+^ T cells in tumor immunity (Figure 5), new discoveries of potent subsets within the CD4^+^ lineage, and clinical implications of engineering human CD4^+^ T cell subsets with CAR-specificity to extend treatment outcomes.

Cytokine and costimulatory cues can polarize naïve CD4^+^ T cells into distinct subsets, such as Th1, Th2, Th17, Th9, Th22, T follicular helper, and Treg. The presence of various cytokines needed during activation by antigen-presenting cells to generate these various subsets is reviewed elsewhere (129–131). In the context of tumor immunity, CD4^+^ T helper cells aid activation of CD8^+^ cytotoxic lymphocytes (132, 133), but can also eradicate tumors in the absence of CD8^+^ T cells (134, 135). The relative antitumor immunity of Th1, Th2, Th17, and Tregs has been thoroughly studied, and emerging reports on the potency of human CD4^+^CD26^high^ T cells that possess improved migration, persistence, and multi-functionality underscores the rationale for translating adoptive transfer of CD4^+^ T cells clinically (135).

The historical understanding of T helper subsets originated with a hypothesis of two opposing helper subsets, termed Th1 and Th2, with distinct functions in promoting cell-mediated or humoral immunity, respectively (136). While both Th1 and Th2 have demonstrated some degree of antitumor efficacy *in vivo*, Th1 cells were shown to induce a CD8^+^ CTL memory response against antigen rechallenge while Th2 cells did not (137). The mechanism of Th1-mediated immunity relies on their production of IFN-γ, which can augment CD8^+^ T cell infiltration and macrophage production of nitric oxide to induce tumor cell apoptosis (138). In a recent clinical trial, CD4^+^ Th1 cells specific for ERBB2IP were successful in regressing a patient’s metastatic cholangiocarcinoma (127). Conversely, Th2 cells, as producers of IL-4, have largely been regarded to promote tumor growth because they inhibit the Th1 polarization program and produce suppressive IL-10 (139). Other reports reveal that Th2 cells stimulate tumor necrosis through inhibition of angiogenesis (140). Recently, in a prophylactic myeloma model, adoptive transfer of Th2 cells induced a strong type II inflammatory response at the tumor site and prevented tumor growth *via* M2-macrophages producing arginase (141). However, these cells were transferred into a host deficient in IFN-γ, which may itself support persistence of Th2 cells, so translational relevance of their efficacy is debatable. Also, dissent over the role for Th2 cells in ACT is furthered since arginase activity has previously been correlated with tumor progression (142). This body of work underscores a need to further understand the role of Th2 cells in antitumor immunity.

Th17 cells, characterized by high IL-17 production, play a contested role in tumor immunity but have been shown highly potent in several preclinical ACT models. Th17 cells are phenotypically polarized by the cytokines IL-6, TGF-β, and IL-1β *via* induction of STAT3 and RORγT and are maintained long term by IL-21 and IL-23 (143–146). ICOS costimulation fosters differentiation and expansion of Th17 cells (147) as well as the function of IL-17-producing CD8^+^ T cells (148). Incorporation of an ICOS costimulatory domain in CAR T cells augments persistence of co-adoptively transferred CD8^+^ T cells in a humanized model of mesothelioma (60). In several different cancer models, transfer of Th17-polarized cells enhanced survival and tumor regression superiorly to Th1 or unpolarized CD4^+^ cells (147, 149, 150). In addition, when expanded *ex vivo* long term, Th17 cells retain their antitumor efficacy while Th1 cells lose tumor control (151). Phenotypically, Th17 cells express more stem-like markers (CCR7, Lef1, TCF7) and fewer exhaustion markers (PD-1, KLRG-1, Tim3) compared with their Th1 counterparts, possibly contributing to longevity (150, 151). Important to the field of CAR therapies, Th17-polarized human meso-CAR T cells exhibit enhanced immunity against mesothelioma versus Th1-polarized cells after both short and long expansion (151). Also, in patients with CLL treated with CD19-CAR T cells, complete responders had CAR T cells with a transcriptomic profile of STAT3/IL-6 signaling, generating a type-17 signature with higher production of IL-17 and IL-22 compared with non-responders (152). Thus, it is truly possible that isolating human PBMCs and polarizing cells to a Th17 phenotype during CAR transduction and activation may generate a therapy with enhanced persistence and thus a long-lived response in patients with solid tumors refractory to treatment with standard bulk CAR T cell preparations.

Despite such preclinical evidence of antitumor potency, adoptive transfer of engineered cells polarized to Th17 phenotype has not been translated yet clinically. The numerous cytokines required to polarize may generate a T cell with enhanced stem-like properties and persistence but also represent a major hurdle halting ease of translation. Our lab has recently described a novel method for isolating potent CD4^+^ T cells *via* surface expression of CD26, an ectoenzyme with costimulatory properties (130, 135). In work pioneered by Nelson and Bailey, CD4^+^ T cells expressing high levels of CD26 are polyfunctional, secreting up to five cytokines simultaneously including IL-17 and IFN-γ, and have robust migratory capacity. CD4^+^CD26^high^ meso-CAR T cells are highly potent against difficult to treat mesothelioma and pancreatic tumors, and have superior persistence compared with other subsets expressing intermediate or low levels of CD26 (135). Clinical translation of CAR-engineered CD26^high^ cells could support superior trafficking, long-term persistence and cytotoxicity at baseline, which could be further enhanced with fourth-generation CARs; thus, these cells are a strong candidate for overcoming major barriers to successful solid tumor CAR therapies.

T cell memory, persistence, and therapeutic efficacy are tightly related to metabolic state, and within an unfavorable environment such as a solid tumor, their ability to use nutrients for energetic needs may mean the difference between life and death of the cell. Just as different memory or helper subsets have varied capacity to kill solid tumors, T cells armed with a superior metabolic state are more equipped to exert their effector functions and generate long-lasting memory responses against tumor antigens. Therefore, we present metabolic manipulation of antitumor T cells as another approach to generating potent therapies below.

### Fine-Tuning Metabolic Fitness

How T cells use energy to survive in the tumor microenvironment has recently gained the interest of cancer immunotherapists. Manipulation of T cell bioenergetics to elicit immunity to solid tumors has shown great promise recently in the preclinical setting. At the fundamental level, it is now clear that lymphocytes engage specific metabolic pathways to best support their functions, intricately regulated by nutrient demand and availability (153). Resting T cells favor energy production through the TCA cycle and fatty acid oxidation (154). Once activated, however, both CD4^+^ and CD8^+^ T cells become quickly poised to exert an effector response, and thus upregulate biosynthetic pathways and rely on aerobic glycolysis, where glucose is rapidly consumed and shuttled through glycolysis to lactate to support their proliferation and effector functions (154, 155). Conversely, memory and Treg cells operate using mitochondrial metabolism and fatty acid β-oxidation in a similar manner as naïve T cells (156). Induction of anabolic, glycolytic pathways may augment proliferation and the inflammatory nature of T cells but correlates with poorer persistence *in vivo*, which in adoptive transfer therapy directly associates with a less effective antitumor response (157, 158). As memory-like T cells are most effective in mediating long-term responses to solid tumors, new data implicates that modulation of their metabolism to favor catabolic pathways may generate a lymphocyte population with enhanced antitumor functions *in vivo*.

Yet, complete denial of anabolic pathways is not a quality of successful T cell therapies. In fact, blunting the anabolic pathway in T cells prevents their capacity to lyse targeted antigens. For example, in models of autoimmunity, genetic deletion of *Glut1* prevented effector T cells from causing pathology in inflammatory bowel disease (159). Inhibition of fatty acid synthesis in T cells limited Th17-induced autoimmunity and promoted a Treg signature, notoriously implicated in promoting tolerance to tumors (160). Pharmacologic inhibition of glycolytic enzyme GAPDH with dimethyl fumarate prevented acquisition of effector function in Th17 cells and skewed their polarization *ex vivo* toward a Treg phenotype, reducing autoimmune pathology in models of experimental autoimmune encephalitis (161). Therefore, direct inhibition of glycolysis in T cells is likely to be deleterious for cancer therapies. Augmentation of fatty acid oxidation in CD8^+^ T cells by treating mice with metformin, on the other hand, promoted memory T cell formation and enhanced immunity to tumor challenge post vaccination (162). Fostering a balance between memory-like metabolism and intrinsic support of glycolysis in CAR T cells may be important for maintaining T cell function and fate within the metabolically restricted tumor microenvironment when the supply of glucose and oxygen is limited (163).

Interestingly, several groups have demonstrated that metabolic manipulation of T cells *in vitro* can benefit antitumor efficacy of transferred cells *in vivo*. Overexpression of glycolytic enzyme phosphoglycerate-mutase 1 limited persistence of transferred CD8^+^ T cells, while inhibition of glycolysis with 2-deoxyglucose augmented stem memory characteristics like Tcf7 and Lef1 expression, and significantly enhanced survival of tumor-bearing hosts (164). Inhibition of AKT signaling, discussed previously as a method for reducing T cell differentiation *ex vivo*, was also shown to decrease glycolytic function and enhance mitochondrial spare respiratory capacity in CD8^+^ T cells (165). Moreover, when these AKTi-treated T cells were transferred into mice, they persisted superiorly to untreated cells (165). Similarly, inhibition of the inositol triphosphate receptor, an important second messenger for calcium release from intracellular storage, in CD4^+^ T cells *ex vivo* prevented glycolytic initiation due to altered calcium flux, fostered a CM phenotype, and augmented their therapeutic efficacy against established melanoma tumors (166). Interestingly, the integrity of the mitochondria in T cells also profoundly impacts their capacity to mount durable immunity to tumors. For example, Pearce and colleagues showed that mitochondrial morphology is tightly related to T cell metabolism; fused mitochondria, described as tubular and closely associated, were characteristic of memory T cells. Conversely, effector T cells were composed of “fissed” or distinct mitochondria dispersed throughout the cytoplasm (167). Forced mitochondrial fusion and inhibition of fission of T cells *via* pharmaceutical approaches *ex vivo* using M1 and Mdivi-1, respectively, promoted a superior antitumor response once transferred *in vivo* (167).

Emerging data reveal that programming and polarization of CD4^+^ T cells also critically determines metabolic commitments and modulates their antitumor properties. Recently, the Mehrotra lab reported that *ex vivo* polarized Th1/Th17 hybrid cells upregulate glutaminolysis and rely on oxidative phosphorylation compared with glycolytic-Th1 cells, ultimately supporting their superior antitumor capacity over traditional Th1 or Th17 cells (168). Homeostatic gamma chain cytokines have also been shown to alter the metabolic fate of antitumor T cells. For example, priming T cells with IL-15 (169) or IL-21 (170), previously discussed as a potential method for enhancing their stemness, redirects metabolism away from glycolysis in favor of fatty acid β-oxidation. This bioenergetic signature directly correlates with T cells possessing longer-lived memory responses to tumors and foreign antigen. Thus, it seems that holding back acquisition of full effector glycolytic capacity in CAR T cells *ex vivo* before infusion could greatly enhance persistence of cells in patients, augmenting therapeutic outcomes in solid tumors.

## Impact of Host Immunity

It is possible that targeting solid tumors *via* a single or combination of several known surface antigens, even with the most persistent or metabolically fit T cell subset, will not be sufficient to evoke cures in patients with heterogenous hard-to-treat solid tumors. Thus far, TIL therapies and ICB have shown greater responses in treating these types of tumors, likely through their ability to induce or bolster an endogenous response of exhausted cells against a highly personalized repertoire of neoantigens existing within the tumor (171–173). TIL therapies in melanoma have shown response rates of up to 50% in contrast to previously FDA approved therapies such as interleukin-2 with response rates near 15% at best (174), and melanoma patients with the highest neoantigen load have the best progression-free survival (175). PD-1 blockade success in clinical trials has led to FDA approval for solid tumors such as metastatic melanoma, advanced NSCLC, recurrent or metastatic SCC of head and neck, refractory classical Hodgkin lymphoma, urothelial carcinoma (176), and as second line in MMR/microsatellite instability-high tumors as of May 2017.

As TIL therapies and checkpoint blockade have generated robust results in patients in several solid tumors, it is likely that incorporating the mechanisms of TIL/ICB into CAR constructs may improve their efficacy. Activated CAR T cells within the tumor microenvironment do express high concentrations of exhaustion markers such as PD-1, Tim-3, Lag3, and 2B4 (177). PD-1 expression also contributes to reduced efficacy of transferred cells regardless of tumor specificity (177). Strategies to improve efficacy of PD-1 expressing, exhausted CAR T cells or to rejuvenate host tumor-specific exhausted T cells along with CAR therapy are threefold: (1) genetic removal of PD-1 from CAR T cells, (2) combination PD-1 blockade with CAR infusion, or (3) CAR T cell production of PD-1 blockade within the host. These strategies and our recommendations for designing next-generation CAR therapies with highest efficacy are discussed below.

The first evidence of reducing PD-1 signaling from a CAR T cell was shown through a PD-1 dominant negative receptor, where engagement of PD-1/PD-L1 would not generate a signal (178). The dominant negative receptor enhanced the functionality of CAR T cells and survival of mice treated with meso-CAR against mesothelioma compared with control CAR with the ability to signal PD-1 (178). Recent advances in genome editing using CRISPR/Cas9 technology have permitted removal of PD-1 entirely from T cells, and in two solid tumor models (prostate and glioma) have shown benefits of this intervention for tumor regression (179, 180). While important for efficacy of transferred cells, and likely to be incorporated into more T cell therapies in the near future, removal of PD-1 would not benefit endogenous exhausted cells specific for potentially unknown antigens. In addition, genetic deficiency of PD-1 has been shown to induce terminally exhausted cytotoxic CD8^+^ T cells; without PD-1, T cells have robust cytokine production and proliferation upon early exposure to antigen, but contract more rapidly and have compromised long-term survival compared with T cells with normal PD-1 expression (181). Thus, genetic removal of PD-1 may not benefit CAR T cell survival long term.

**Figure 6:**
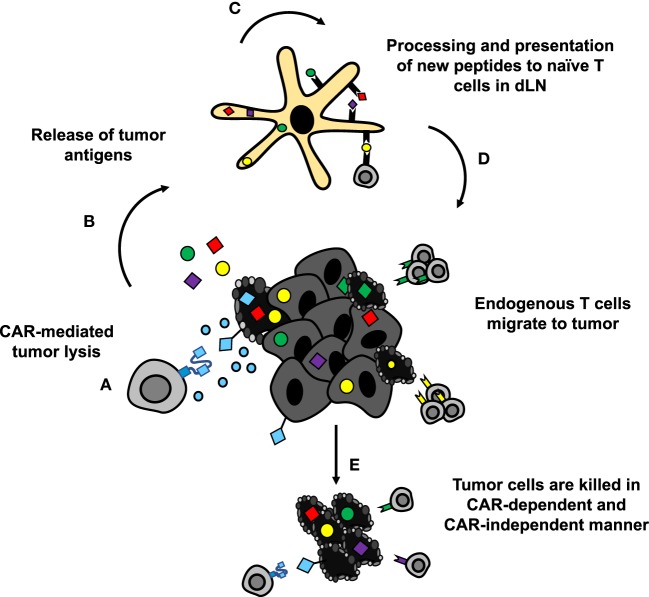
CAR-mediated tumor destruction can synergize with host immunity through epitope spreading. **(A,B)** Chimeric antigen receptor (CAR)-mediated tumor cell lysis induces inflammation, and release of tumor antigens. **(C)** DAMPs from dying cells recruit APCs to tumor site, which take up and process the released antigens for presentation. **(D)** APCs present newly processed tumor antigens to naïve T cells in lymph nodes. Activated T cells migrate to the tumor site. **(E)** Tumor-specific lymphocytes synergize with CAR T cells to eradicate difficult to treat solid tumors.

Theoretically, CAR-mediated destruction of tumor cells could also lead to generation of new antigen-specific lymphocytes *via* epitope spreading (Figure 6) (182). These newly activated cells are susceptible to suppression within the tumor similar to CAR T cells. To overcome this limitation, PD-1 blockade could be given in combination with or could be encoded by CAR T cells to both support the transferred cells and the endogenous tumor-specific lymphocytes. Preclinically, combination therapies in solid tumors have demonstrated enhanced proliferation, function, and antitumor efficacy of HER2-CAR T cells in breast cancer and sarcoma (183). At the time of writing, clinical trials with such combinations are heavily skewed toward blood cancers (NCT02926833, NCT02706495, and NCT03287817 in DLBCL, NCT03310619 in B cell NHL, and NCT02650999 in DLBCL, follicular lymphoma, and mantle cell lymphoma; ClinicalTrials.gov identifiers). Preliminary results in these hematologic malignancies suggest that PD-1 blockade may enhance CAR T cell persistence and could improve objective responses in patients (184, 185). Thus, there is rationale for combining these approaches to improve persistence of CAR T cells and generate more robust responses.

This principle could be streamlined even further if the CAR T cells produced monoclonal antibodies that inhibit checkpoint molecules themselves. Preclinical evidence in lung and ovarian tumors shows that CAR T cells producing PD-1 blocking antibodies are more therapeutic than control CAR T cells against the same target (186). Importantly, CAR T cell production of PD-1 antibody was more effective than systemic administration of the antibody, which could be related to localized, high dose delivery (186). Similar results were found in a renal cell carcinoma model where production of antibodies to PD-L1 enhanced CAR T cell function, though the results were less dramatic (187). Since both of these studies were conducted in NSG mice, the efficacy of ICB-producing CAR T cells may be even more striking in a host with an intact immune system. These preclinical results were rapidly translated to several clinical trials in China for variety of solid tumors, described in Table 1.

## The Ultimate Car T Cell Therapy

**Figure 7:**
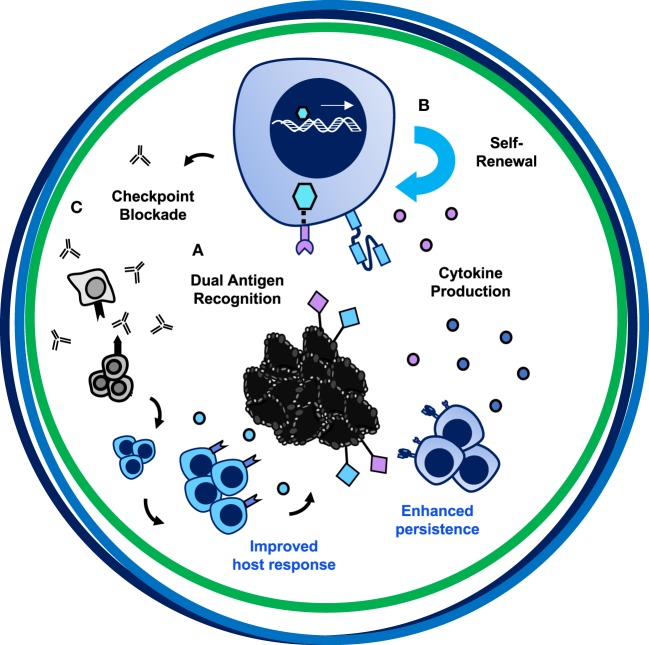
The trifecta of successful chimeric antigen receptor (CAR) T cell therapies in solid tumors. The ultimate CAR T cell therapy has tumor specificity, potent migratory capacity and persistence, and improves the host immune response. **(A)** Bispecificity through syn-Notch technology augments targeting to tumor/tumor-specific tissue. **(B)** Engineering a T cell with enhanced persistence and migratory capacity—such as a Th17 or CD4^+^CD26^high^ cell—or with self-renewing properties—such as a CD8^+^ T_SCM_ cell—will enhance long-term memory responses to prevent tumor recurrence. **(C)** Secretion of PD-1 blockade and cytokines such as IL-12, IL-15, IL-18, or IL-21 locally could overcome the suppressive tumor microenvironment, reinvigorate the exhausted host immune response to other tumor antigens, and synergize with CAR-specific T cells to destroy large heterogenous solid tumors.

Chimeric antigen receptor T cell therapies exemplify an incredible opportunity—one like Hippocrates described—to take control of healing patients through empowering and redesigning a patient’s own T cells to destroy tumor cells. As depicted in Figure 1, for a CAR T cell therapy to be more successful in solid tumors, the design should encompass three axes. Illustrated in Figure 7, we posit that this therapy would incorporate bispecificity through the CAR construct, generate enhanced potency *via* engineering a superior T cell subset, and revitalize the host immune response through cytokine and checkpoint antibody secretion. First, to enhance specificity, syn-Notch inducible CAR expression upon engagement of a tissue-specific antigen could improve sensitivity of the CAR to target the tissue and reduce off-tumor effects. Secondly, to enhance persistence, trafficking, and self-renewal properties, a CAR-engineered T_SCM_ CD8^+^ T cell expanded with pharmacologic inhibitors or generated from iPSCs, or either a Th17 cell or a CD4^+^CD26^high^ T cell could overcome these limitations of poor quality T cell infusion products. Use of a multipotent T cell may permit adoptive transfer of fewer cells, thereby streamlining and reducing the cost and time investment to generate T cell products for infusion. A lower dose of T cells could reduce risk for severe toxicities and cytokine storms; however, engineering such a potent cell could alternatively be more toxic to patients when infused. Therefore, safety switches or Boolean logic gates should be incorporated to prevent life-threatening adverse events. Finally, taking advantage of the host’s response to personalized neoantigens, PD-1 antibody-producing CAR T cells that also produce cytokines like IL-12, IL-15, IL-18, or IL-21 locally in the tumor after engaging a tumor-specific antigen would counteract the highly suppressive environment and synergize the power of the endogenous immune response with the genetically redirected CAR T cell response.

Though the combination of these approaches is theoretically appealing, a T cell incorporating the several mechanisms proposed has not yet been engineered. Such a construct may prove difficult to generate without interruption of normal gene function. Use of CRISPR/Cas9 could direct incorporation to a specific location in the genome to enhance efficacy, such as the *TRAC* locus (188). Generating a universal CAR that is not MHC restricted, where infusion products could be mass-produced versus individually developed for each patient, could also make this CAR design feasible. The ease of developing a T cell as proposed is likely to improve over time as academic and industrial facilities expand and commercial-grade production becomes streamlined through automation and improved quality control (189). While it may make for a complex construct, harnessing capabilities of genetic redirection, optimal T cell subsets, and augmented crosstalk to other infiltrating immune cells may be one attainable approach to eradicate heterogenous and therapeutically resistant solid tumors.

## Conclusion

Adoptive cell transfer with CAR-redirected T cells is a potentially curative approach for patients with previously treatment-resistant tumors. CAR T cells have proven their potency against hematologic cancers evidenced by their recent FDA approvals for B-ALL and DLBCL. For solid tumors, these therapies remain in early development but may require a new approach to enhance their efficacy. Herein, we have presented a combinatorial approach to augment the ability of CAR T cells to overcome challenges they face within the tumor microenvironment. We posit that a future CAR T cell armored with (1) a superior targeting system specific to the tumor and tumor tissue, (2) engineering of a highly potent, persistent, and self-renewing T cell subset, and (3) rejuvenation of the endogenous host response through CAR T cell production of monoclonal antibodies against immune checkpoint molecules will bolster the immune attack on the solid tumor to best reduce toxicity and support a long-lived memory response against targeted antigens and personalized neoantigens. Elegant findings from investigators worldwide will continue moving forward the solid tumor CAR T cell approach to generate cures for patients with previously therapeutically resistant cancers.

## Author Contributions

HK and CP conceptualized, wrote, and edited the manuscript. AS, CD, MW, and SM provided feedback and edited the manuscript.

## Conflict of Interest Statement

The authors declare that the research was conducted in the absence of any commercial or financial relationships that could be construed as a potential conflict of interest.
